# Multiplex IgE peanut panels: a critical appraisal of assay designs and the good, the bad, and the ugly features of the applied allergen components

**DOI:** 10.3389/falgy.2025.1515294

**Published:** 2025-06-02

**Authors:** D. de Boer, C. J. J. Bijnens, M. C. Slot, C. M. G. Nieuwhof, J. A. P. Bons

**Affiliations:** ^1^ Central Diagnostic Laboratory, Maastricht University Medical Center+, Maastricht, Netherlands; ^2^ Department of Allergology and Clinical Immunology, Internal Medicine, Maastricht University Medical Center+, Maastricht, Netherlands

**Keywords:** multiplex assay, specific IgE, peanut, allergen component, isoallergen, allergen variant

## Abstract

**Background:**

Multiplex allergy assays are currently well-established in allergy diagnostics. However, the different assays in terms of designs and performance are also claimed to be heterogeneous as no agreed standards and requirements are available.

**Objective:**

We aimed to compare the analytical assay designs of the ISAC, ALEX, and EUROLINE peanut (Ara h) panels and the features of the applied isoallergens and variants to create more awareness of the heterogeneity of multiplex allergy assays.

**Methods:**

We conducted a multi-source survey in publicly available data sources and among manufacturers and performed correlation studies using patients’ serum samples.

**Results:**

The survey proved that the panels are indeed very heterogeneous in many ways, especially regarding the allergen component origin and isoallergen composition. Despite that, we found adequate correlations between IgE against the clinically relevant Ara h storage proteins measured by the panels. However, for the clinically relevant lipid transfer protein Ara h 9, the correlations were less adequate, which could be caused by the different Ara h 9 isoallergens used in the studied panels. For cross-reactive carbohydrate determinants (CCDs), the results were complicated, which also corresponds to the complex nature of CCDs and the different inhibition procedures. The detection of subpopulations of patients for all panallergens illustrated the heterogeneous nature of peanut IgE in general and of the peanut panels studied. Regarding the overall features provided for the three panels, we classified the peanut allergen components and CCDs by their good, bad, and even ugly features when used within these panels.

**Conclusions:**

Knowledge of the origin and respective isoallergen specifications of the peanut allergen components including the exact CCD composition is essential. Together with that of the variants, this should be documented more adequately in scientific studies and in the respective instructions for the use of multiplex allergy assays.

## 1 Introduction

Multiplex approaches are *in vitro* methods that simultaneously analyze specific immunoglobulin Es (sIgEs) against different allergen components. These methods are becoming well-established (1–5). In general, multiplex assays are presented as semi-quantitative assays and are meant principally to reveal broad patterns of IgE-mediated sensitizations. In this way, the identification of causative allergens and potential allergic cross-reactivity contribute to the diagnosis and management of allergy (1, 3).

While multiplex allergy assays have been designed to overcome certain restrictions of quantitative singleplex assays, the interpretation of the results of multiplex assays is complicated (3). Besides this, simultaneously analyzing different sIgEs in one assay is itself problematic and has created several analytical challenges. As no commonly agreed standards and requirements for multiplex assay are available and companies want to put a unique product on the market, every manufacturer of commercial multiplex assays is dealing with these problems and challenges in their own way. Consequently, the different multiplex allergy assays are heterogeneous in terms of design and performance. Because of this, comparing and evaluating these assays is even more challenging.

Obviously, manufacturers decide themselves which allergen extracts or components are part of their multiplex allergy assay. However, with respect to the allergen and component nomenclature, there should be uniformity and transparency. Regarding the allergen component nomenclature, commercial multiplex assays partially use the systematic World Health Organization and International Union of Immunological Societies (WHO/IUIS) nomenclature for allergen names to specify their applied allergen components (6). Another applied nomenclature in the commercial assays is the biochemical function designation to the components, resulting in a classification of protein families containing cross-reactive panallergens [e.g., storage proteins, lipid transfer proteins (LTPs), pathogenesis-related proteins-10 (PR-10) and profilins]. Seldom applied are WHO/IUIS definitions such as isoallergen and variant. As commercial multiplex assays do not specify these terms regarding their allergen components, the exact nature of the applied allergen components (e.g., origin and isoallergen and variant composition) to bind and detect sIgE remains unknown for users of these assays. While this is apparently not of concern to most users of multiplex assays, it deserves more attention, especially since the features of the components determine the diagnostic interpretation.

The goal of this study is to examine some critical assay characteristics of sIgE against peanut allergen components and compare the analytical assay designs of the ISAC, ALEX, and EUROLINE peanut panels and the features of applied peanut isoallergens and variants. In this way, we aim to create more awareness among those who manufacture and use multiplex assays in allergy diagnosis and management. Above all, adequate transparency and a better understanding of the complexity of multiplex assays are essential to estimate and appreciate the clinical impact that multiplex IgE peanut panels may have.

## 2 Materials and methods

### 2.1 Clinical samples

All clinical samples were left-over serum samples and were collected in the context of allergy *in vitro* diagnostics. Initial sIgE concentrations were screened for peanut sensitization either by the ImmunoCAP, ISAC, or EUROLINE assays. The handling of samples was in accordance with the code for proper use in the Netherlands and consisted of anonymization and collection of the sIgE concentration data (7).

### 2.2 Assessment and comparison of assays

Different multiplex allergen assays were subjected to a critical assessment regarding the overall analytical design and the composition of isoallergens and the variants of their peanut allergen components [ISAC112 (Thermo Fisher Scientific), ISAC E112i (Thermo Fisher Scientific), EUROLINE DPA-Dx peanut 1 (EUROIMMUN Medizinische Labordiagnostika), ALEX^1^ (Macro Array Diagnostics), and ALEX^2^ (Macro Array Diagnostics)]. ISAC112 preceded ISAC E112i and ALEX^1^ preceded ALEX^2^. Information was obtained from different sources, either from the literature and instructions for the use of the assays or directly from the manufacturer upon request. An isoallergen is defined in this context as an allergen from a single species, sharing similar molecular size, identical biological function, and ≥67% amino acid identity. Variants are multiple forms of highly identical sequences (>90% identity, typically differing in only a few amino acids) of one isoallergen, which are thus designated as variants of that isoallergen (6). The term “natural allergen” is used to indicate any allergen purified from natural source material, while “recombinant allergen” is produced in bacterial, yeast, or mammalian expression systems. Natural allergens, which are sometimes also designated as native allergens, are denoted by the prefix “n” to distinguish them from recombinant allergens, which are indicated by the prefix “r” before the allergen name. Although not currently relevant for peanut allergen components, synthetic peptides are indicated by the prefix “s.” Isoallergens and variants are denoted by the addition of four numerical suffixes to the allergen name. The first numerals distinguish between isoallergens and the last two between variants, e.g., PR-10 protein of *Arachis hypogaea*: allergen name Ara h 8, WHO/IUIS recognized isoallergens Ara h 8.01 and Ara h 8.02, and corresponding single variants Ara h 8.0101 and Ara h 8.0201.

Serum samples from patients who were screened routinely for peanut whole extract (ImmunoCAP) or peanut allergen component sIgE (ISAC or EUROLINE) were considered (Supplementary Material S1). Samples were included in the first step if screening results indicated at least one positive result for peanut sensitization. Inclusion criteria were at least one positive result for the ImmunoCAP (≥0.35 kU**_A_**/L), ISAC (≥0.3 ISU-E), or EUROLINE (≥0.35 kU/L). Samples that contained left-over volume >150 µL were included in the second step (*N* = 74). If the aliquot volume was >200 µL, then the series of multiplex assays were completed up to three assays (*N* = 34; ISAC, EUROLINE, and ALEX). If the aliquot volume was <200 µL, only the ISAC and EUROLINE panels were performed (*N* = 40; ISAC, EUROLINE). All assays were performed according to the manufacturer's instructions. For the EUROLINE assay, the overnight incubation version was chosen to minimize the usage of sample volume.

### 2.3 Result management and statistical analysis

Concentrations of sIgE were graphically displayed in scatterplots using SigmaPlot (Systat Software). All measured concentrations were treated as quantitative results despite the fact that the ISAC and EUROLINE panels were presented by their manufacturers as semi-quantitative assays (Table 1).

**Table 1:** Assay characteristics of the multiplex allergy assays ISAC112, ISAC E112i, EUROLINE DPA-Dx peanut 1 panel, ALEX^1^, and ALEX^2^.

Assay characteristic	ISAC112 and ISAC E112i	EUROLINE peanut panel	ALEX^1^	ALEX^2^
Number of targets in a single run	112 allergen components No natural extracts	Nine allergen components No natural extracts	155–156 natural extracts 125–127 components (the exact number depends on subversions of ALEX^1^)	118 natural extracts including one mix of three natural extracts (a total of 120 extracts) 182 components including two mixes of two components (a total of 184 components) Five components research-use-only
Required sample volume	30 µL	100 µL (overnight incubation) 175 µL (2 h incubation) 400 µL (1 h incubation)	100 µL	100 µL
Analytical status according to claims of the manufacturer	Semi-quantitative for specific IgE No quantitation for total IgE	Semi-quantitative for specific IgE No quantitation for total IgE	Quantitative for specific IgE Semi-quantitative for total sIgE	Quantitative for specific IgE Semi-quantitative for total sIgE
Detection technology	Fluorescence spectroscopy	Colorimetry	Colorimetry	Colorimetry
Unit for calculated concentration	ISU-E	kU/L	kU_A_/L	kU_A_/L
Calibration of specific IgE	On-site homologous calibration Four specific IgE concentrations, recombinant specific IgE against 14 components A concentration range of 0.5–50 ISU-E reporting range of specific IgE 0.3–100 ISU-E	Off-site semi-continuous curve reporting range 0.35–100 kU/L	On-site heterologous calibration six total IgE concentrations concentration range 0.3–50 kU_A_/L reporting range of specific IgE 0.3–50 kU_A_/L	On-site heterologous calibration Six total IgE concentrations concentration range 0.3–50 kU_A_/L reporting range of specific IgE 0.3–50 kU_A_/L
Reference preparation for total IgE	Directly traceable (via an unbroken chain of calibrations) to the second IRP 75/502 (equivalent to the 3rd International Standard 11/234) of human serum immunoglobulin E from the WHO	No information in the instructions for the use of EUROLINE DPA-Dx peanut 1	Indirectly traceable via ImmunoCAP (Thermo Fisher Scientific) to the second IRP 75/502 of human serum immunoglobulin E from the WHO	Indirectly traceable via ImmunoCAP (Thermo Fisher Scientific) to the third IRP 11/234 of human serum immunoglobulin E from the WHO
LLoR	0.3 ISU-E for specific IgE	0.35 kU/L for specific IgE	0.1 kU_A_/L for specific IgE	0.1 kU_A_/L for specific IgE
ULoR	100 ISU-E for specific IgE	100 kU/L for specific IgE	50 kU_A_/L for specific IgE	50 kU_A_/L for specific IgE

Concentrations below the lower limit of reporting (LLoR) were represented as LLoR/√2 (8), while those higher than the upper limit of reporting (ULoR) were represented arbitrarily as ULoR×√2 (for assay-specific LLoRs and ULoRs, see Table 1). Analytical correlations between sIgE concentrations against peanut extract or peanut allergen components of the different panels were expressed as R^2^_adjusted_ and calculated using SigmaPlot if an adequate number of observations was available above the LLoR (*N* ≥ 10). For those in which the number of observations was *N* < 10, a visual inspection was performed. Like R, the correlation coefficient, and R^2^, the coefficient of determination, the R^2^_adjusted_ is also a measure of how well a regression model describes the data, but R^2^_adjusted_ takes into account the number of independent variables, which reflects the degrees of freedom. Correlations with absolute R^2^_adjusted_ values closest to 0.7 were classified as strong, closest to 0.5 as moderate, and closest to 0.3 as weak.

A subpopulation detected by a panel was defined as a group consistently detected by one panel and not by the other. It was considered significant if the relative number expressed as a percentage of those values above the respective LLoR was >15%.

## 3 Results

### 3.1 Overall analytical designs of multiplex assays

The analytical assay designs included different solid phases to couple and fix allergen components onto a surface and in this way bind and detect sIgE in blood specimens. The ISAC panel is a chip based on a microscope glass slide coated with a polymer used to couple allergens in an identical way. EUROLINE panel is a line blot strip with dedicated membrane areas used to couple allergens in an optimized way for each allergen. ALEX panel is a membrane in a cartridge that uses dedicated nano-bead technology to couple allergens embedded on a nitrocellulose membrane in an identical way (2, 5). Other characteristics are presented in Table 1.

Required sample volumes varied from 30 µL for ISAC to 100 µL for the ALEX versions and from 100 to 400 µL for EUROLINE; in the EUROLINE assay procedure, the shortest incubation period required the highest and the overnight incubation period the lowest sample volume.

**Figure 1:**
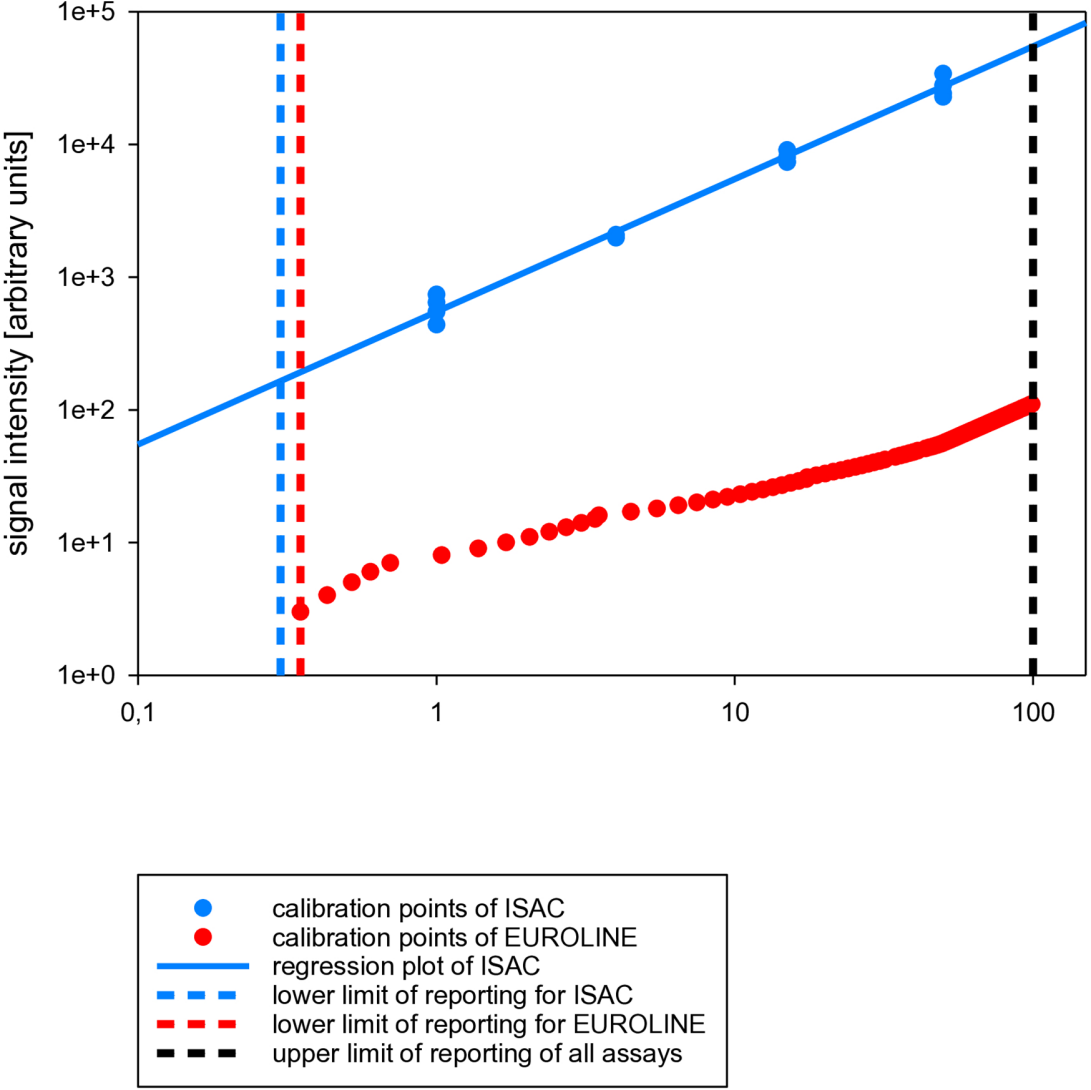
Examples of the calibration curves of ISAC and EUROLINE assays.

The method of calibration was another aspect that was quite different among the multiplex allergy arrays. For example, the calibration of the ISAC112 versions studied was based on an on-site homologous calibration of recombinant sIgE against a selected number of components, the EUROLINE calibration was based on an off-site static calibration curve prefixed in the software, and the ALEX calibration was based on an on-site adjusted heterologous calibration against total IgE. Examples of the calibration curves of the ISAC112 versions and EUROLINE could be retrieved from the respective software, while for the ALEX versions, they could not (Figure 1). The calibration ranges were adjusted to the reporting range, although only for the ALEX versions did this result in a quantitative assay as claimed by its manufacturer. The other two assays were qualified by their manufacturers as semi-quantitative assays.

### 3.2 Allergen component origin and features

All the panels in the study had a Conformité Européene (CE) marking, which is part of the European Union's (EU) harmonization legislation and meant they were deemed to meet EU safety, health, and environmental protection requirements.

In general, the ISAC and ALEX panels are broad-range multiplex assays and are focused not only on the peanut allergen and components but also on a large and comprehensive group of allergens and components other than peanut. In contrast, the EUROLINE panel is a limited panel dedicated to peanut. However, the EUROLINE panel does not exclusively contain true peanut allergen components, but due to commercial availability and other reasons, also so-called surrogate peanut components (for example, Bet v 1 as a surrogate for Ara h 8). In relation to peanut, some components in the ISAC and ALEX panels can also be considered as surrogate peanut components (for example, Bet v 2 as surrogate for Ara h 5), especially since Ara h 5 is not part of the ISAC and ALEX panels. In Table 2, an overview is presented concerning the origin and structure of the true and surrogate peanut components including those of cross-reactive carbohydrate determinants (CCDs), which are applied in the respective multiplex allergen assays.

**Table 2:** Specifications of true and surrogate peanut allergen components including those of CCDs in the multiplex allergy assays ISAC112, ISAC E112i, EUROLINE DPA-Dx peanut 1 panel, ALEX^1^, and ALEX^2^.

Peanut component (PC)	Biochemical designation of PC	Multiplex allergy assays											
		ISAC112 and ISAC E112i			EUROLINE peanut panel			ALEX^1^			ALEX^2^		
*Arachis hypogaea*	WHO/IUIUS	Presence	Ara h isoallergen	Source	Presence	Ara h isoallergen	Source	Presence	Ara h isoallergen	Source	Presence	Ara h isoallergen	Source
Ara h 1	Cupin (vicillin-type, 7S globulin) storage protein	Yes	1.0101	Recombinant (rAra h 1)	Yes	1.0101	Recombinant (rAra h 1)	Yes	Purified from a mixture of isoallergens and variants	Natural extract (nAra h 1)	Yes	Purified from a mixture of isoallergens and variants	Natural extract (nAra h 1)
Ara h 2	Conlutin (2S albumin) storage protein	Yes	2.0101	Recombinant (rAra h 2)	Yes	2.0201	Recombinant (rAra h 2)	Yes	2.0201	Recombinant (rAra h 2)	Yes	2.0201	Recombinant (rAra h 2)
Ara h 3	Cupin (legumin-type, glycinin, 11S globulin) storage protein	Yes	No isoallergen and variant number assigned, but 98% identical to 3.0101	Recombinant (rAra h 3)	Yes	3.0101	Recombinant (rAra h 3)	Yes	Purified from a mixture of isoallergens and variants	Natural extract (nAra h 3)	Yes	Purified from a mixture of isoallergens and variants	Natural extract (nAra h 3)
Ara h 5	Profillin	Bet v 2 as PC surrogate	Not relevant	Recombinant (rBet v 2)	Yes	5.0101	Recombinant (rAra h 5)	Bet v 2 as PC surrogate	Not relevant	Recombinant (rBet v 2)	Bet v 2 as PC surrogate	Not relevant	Recombinant (rBet v 2)
Ara h 6	Conlutin (2S albumin) storage protein	Yes	6.0101	Natural extract (nAra h 6)	Yes	6.0101	Recombinant (rAra h 6)	Yes	Purified from mixture of isoallergens and variants	Natural extract (nAra h 6)	Yes	6.0101	Recombinant (rAra h 6)
Ara h 7	Conlutin (2S albumin) storage protein	No	Not applied in panel		Yes	7.0201	Recombinant (rAra h 7)	No	Not applied in panel		No	Not applied in panel	
Ara h 8	PR-10 protein, Bet v 1 family member	Yes	8.0101	Recombinant (rAra h 8)	Bet v 1 as PC surrogate	Not relevant	Recombinant (rBet v 1)	Yes	8.0101	Recombinant (rAra h 8)	Yes	8.0101	Recombinant (rAra h8)
Ara h 9	Non-specific lipid transfer protein type 1	Yes	9.0101	Recombinant (rAra h 9)	Yes	9.0201	Recombinant (rAra h 9)	Yes	9.0101	Recombinant (rAra h 9)	Yes	9.0101	Recombinant (rAra h 9)
Ara h 15	Oleosin	No	Not applied in panel		No	Not applied in panel		No	Not applied in panel		Yes	15.0101	Recombinant (rAra h 15)
CCD	Cross-reactive carbohydrate determinant	MUXF3 glycan [carbohydrate glycans purified from digested bromelain and coupled to human serum albumin (MUXF3-HSA)]			MUXF3 glycan [carbohydrate glycans purified from digested bromelain and coupled to human serum albumin (MUXF3-HSA)]			Ana c 2 (containing the MUXF3 glycan) and Hom s lactoferrin (containing both the MUXF3 and MMXF3 glycans); a CCD blocker with the MUXF3 glycan coupled to HSA is applied during the sample preparation step			Hom s lactoferrin (containing both the MUXF3 and MMXF3 glycans); a CCD blocker with the MUXF3 glycan coupled to HSA is applied during the sample preparation step		

The exact composition of the isoallergens of a peanut allergen component in the different panels, including in the distinct versions of the same panel, is very heterogeneous (Table 2). In fact, the composition is never exactly identical for any of the peanut components. In the majority of cases, only one recombinant isoallergen is applied, while ALEX sometimes also applies natural mixtures of isoallergens. If one recombinant isoallergen is used, only for the components Ara h 1 and Ara h 6 did the manufacturers select the same isoallergen.

While the peanut allergen components of both successive ISAC versions are identical, those of the successive ALEX versions are not equal. ALEX^2^ contains the oleosin Ara h 15 and the ALEX^1^ does not. Moreover, in the ALEX^1^ panel, Ara h 6 is present from a natural source, while in the ALEX2 panel, it is from a recombinant source. Furthermore, ALEX^1^ includes the peanut whole extract and Ana c 2, which is the glycoprotein bromelain, whereas ALEX^2^ does not. The significance of Ana c 2 is that it can be used as a marker to measure CCDs, especially those directly related to the CCD inhibitor as used in the ALEX versions. The inhibitor consists of carbohydrate glycans purified from digested bromelain and coupled to human serum albumin (MUXF3-HSA), whereas Ana c 2 contains CCDs based on the carbohydrate core MUXF3 (9, 10). Uniquely present in the EUROLINE panel and not in the other panels are the components Ara h 5, which is a profilin, and Ara h 7, which is a 2S albumin storage protein component (Table 2).

### 3.3 Analytical correlations between assay results

#### 3.3.1 Storage protein components

**Figure 2:**
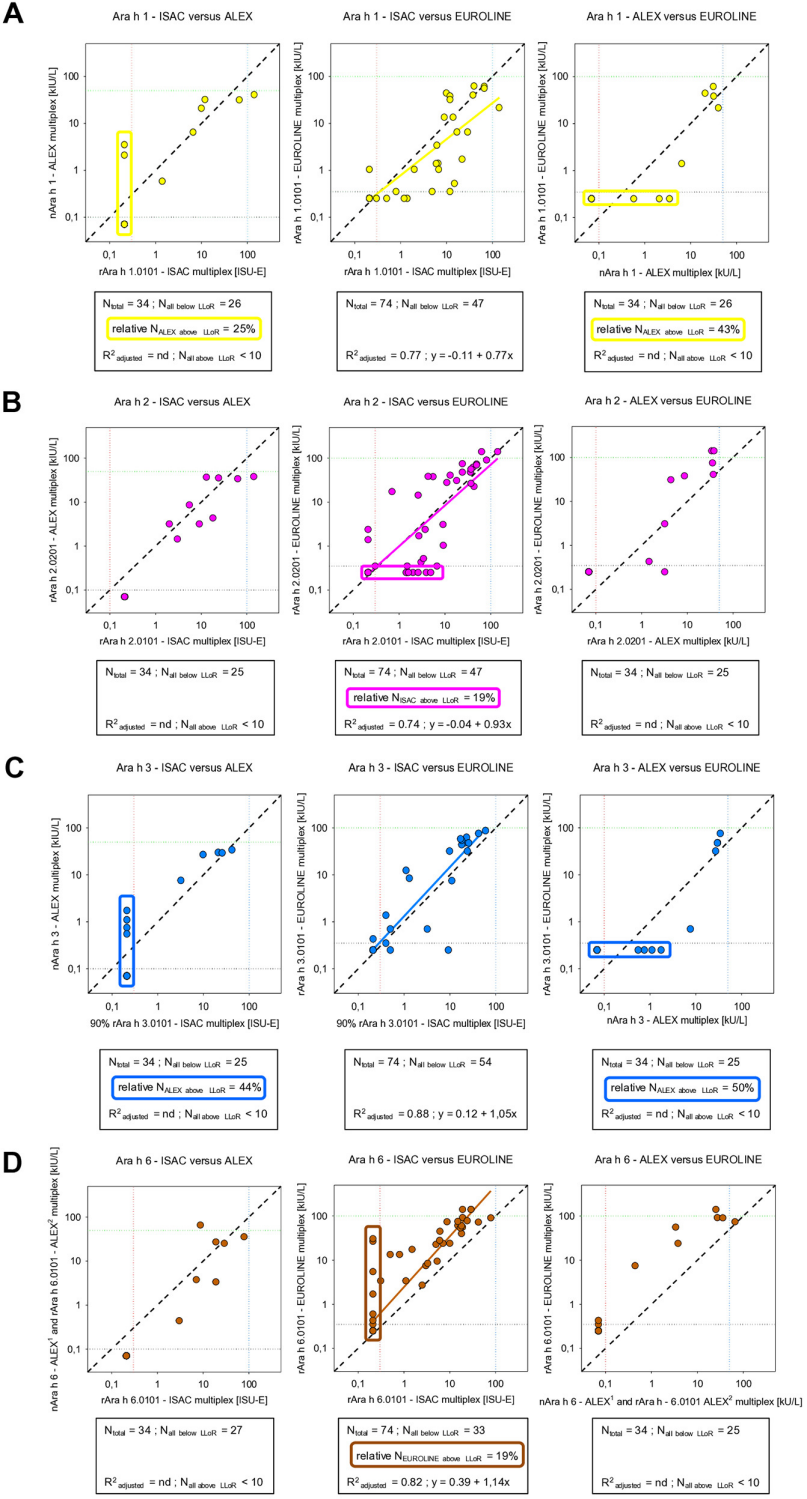
Correlation between concentrations of sIgE against common peanut storage proteins as detected by three multiplex assays: **(A)** Ara h 1 vs. Ara h 1, **(B)** Ara h 2 vs. Ara h 2, **(C)** Ara h 3 vs. Ara h 3, and **(D)** Ara h 6 vs. Ara h 6 (for an explanation of the meaning of the gridlines, see the respective information in Figure 6).

The analytical results concerning sIgE against storage protein components Ara h 1, Ara h 2, Ara h 3, and Ara h 6 correlated strongly (Figures 2A–D), when based on linear correlation (relevant observations *N* ≥ 10). Those based on visual inspection (relevant observations *N* < 10) still correlated adequately, considering the typical observation that the data points are randomly distributed around or close to the line Y = X and the general observation that sIgE against allergens by different multiplex assays never correlate perfectly (11, 12). For sIgE against Ara h 6, the ALEX and ISAC panels produced, in general, relatively low responses, while the EUROLINE peanut panel produced relatively high responses. The ALEX panel seemed to consistently detect additional subpopulations of patients for IgE against Ara h 1 and Ara h 3, which were not detected by the other panels (Figures 2A–C). During the comparison of panels using our larger population study (*N* = 74) the EUROLINE panel detected a subpopulation for Ara h 6, which was not detected in the other panels, as did the ISAC panel for Ara h 2.

**Figure 3:**
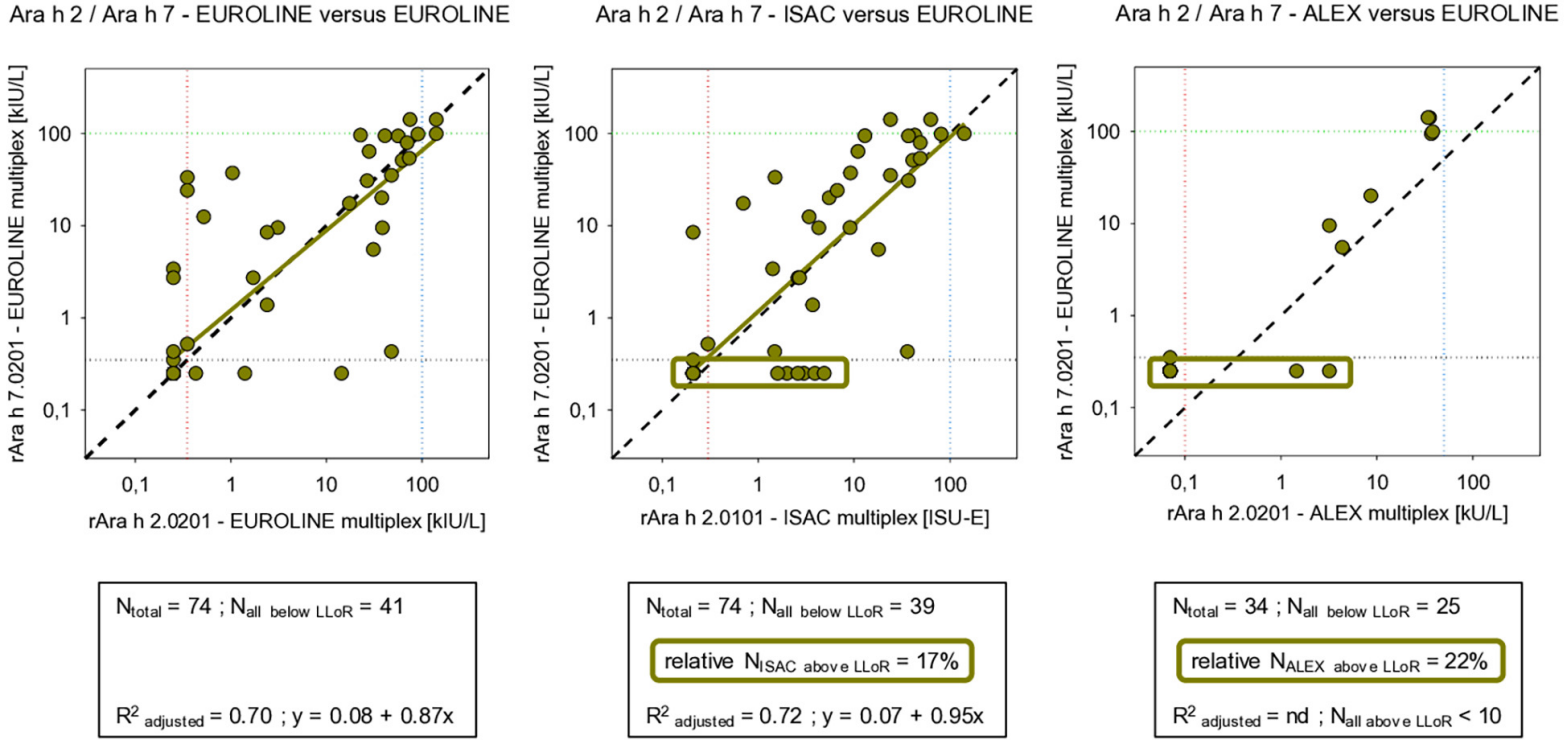
Correlation between concentrations of sIgE against two peanut 2S albumin storage proteins as detected by three multiplex assays. Ara h 2 vs. Ara h 7 (for an explanation of the meaning of the gridlines, see the respective information in Figure 6).

The additional value of measuring IgE sensitization against the storage protein component Ara h 7, as performed by the EUROLINE panel, was limited in the sense that significant IgE mono-sensitization toward Ara h 7 was not observed and was always accompanied by multi-sensitizations against several 2S albumin peanut storage proteins such as Ara h 2 and Ara h 6 (data not shown). Moreover, when correlated with sIgE against Ara h 2 in all three panels, the correlations with that of Ara h 7 in the EUROLINE panel were also strong based on linear correlation or adequate based on visual inspection (Figure 3). The results for Ara h 2 and Ara h 7 also differed as the ISAC panel consistently detected a subpopulation, while ALEX only detected a subpopulation when correlated for Ara h 2.

#### 3.3.2 Lipid transfer protein components

**Figure 4:**
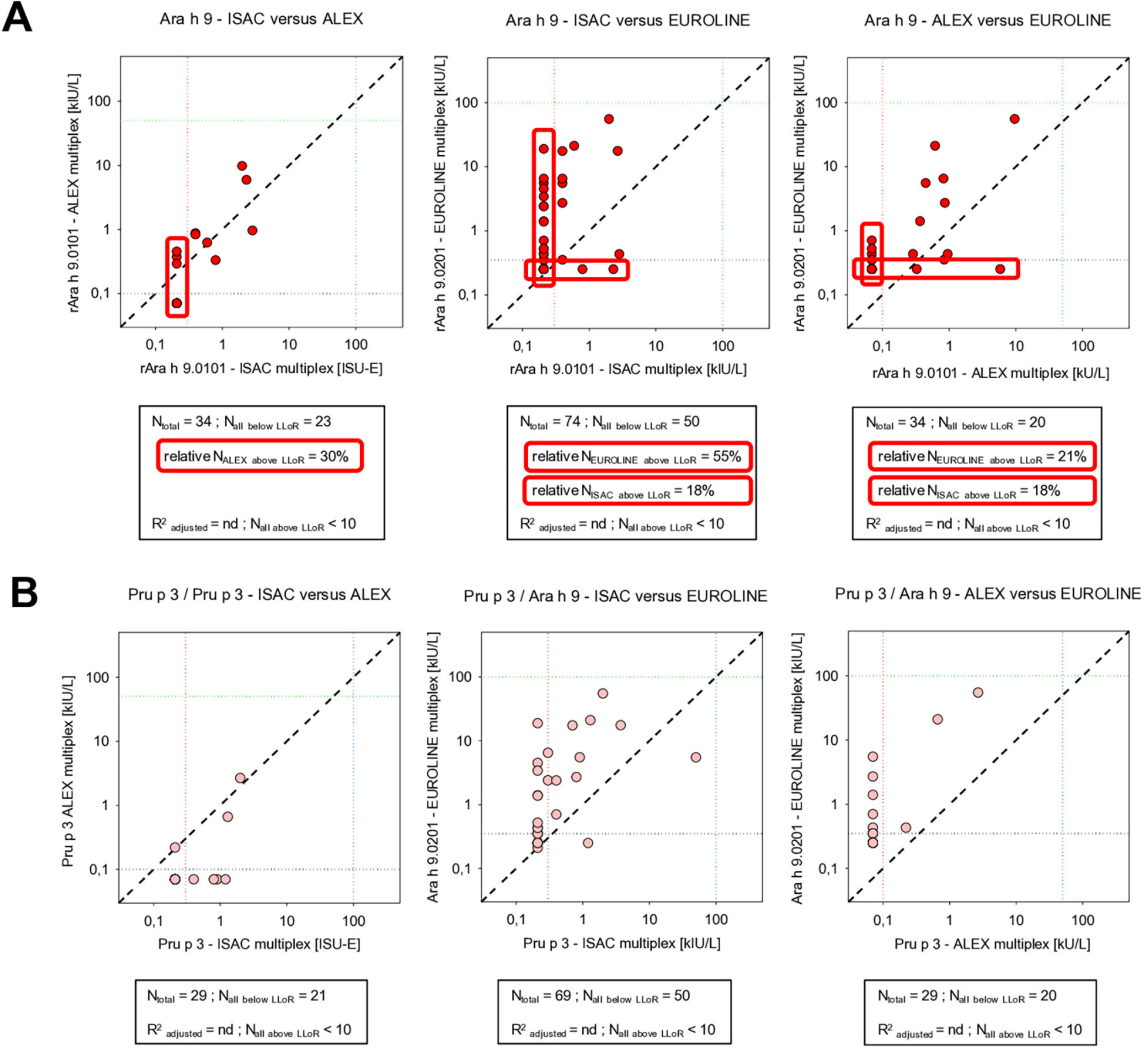
Correlation between concentrations of sIgE against some LTPs as detected by three multiplex assays: **(A)** Ara h 9 vs. Ara h 9 and **(B)** Pru p 3 vs. Pru p 3 or Pru p 3 vs. Ara h 9 (for an explanation of the meaning of the gridlines, see the respective information in Figure 6).

For LTP Ara h 9, the sIgE results of ISAC vs. ALEX and ALEX vs. EUROLINE panel correlated adequately, while those of ISAC vs. EUROLINE did not (Figure 4A). It should be noted that the ALEX and ISAC panels produced, in general, relatively low responses for Ara h 9, whereas the EUROLINE peanut panel produced relatively high responses. These high responses were associated with the presence of the Ara h 9.0201 LTP isoallergen in the EUROLINE panel vs. that of the Ara h 9.0101 isoallergen in the other assays. If the peach LTP allergen component Pru p 3 in ISAC or ALEX is considered as a surrogate LTP allergen component for Ara h 9.0201 in EUROLINE (13), alternative correlations can be examined. However, based on visual inspection, peanut LTP Ara h 9.0201 in the EUROLINE panel also did not correlate adequately with peach LTP Pru p 3 in the ISAC or ALEX panel (Figure 4B).

#### 3.3.3 Surrogate components

**Figure 5:**
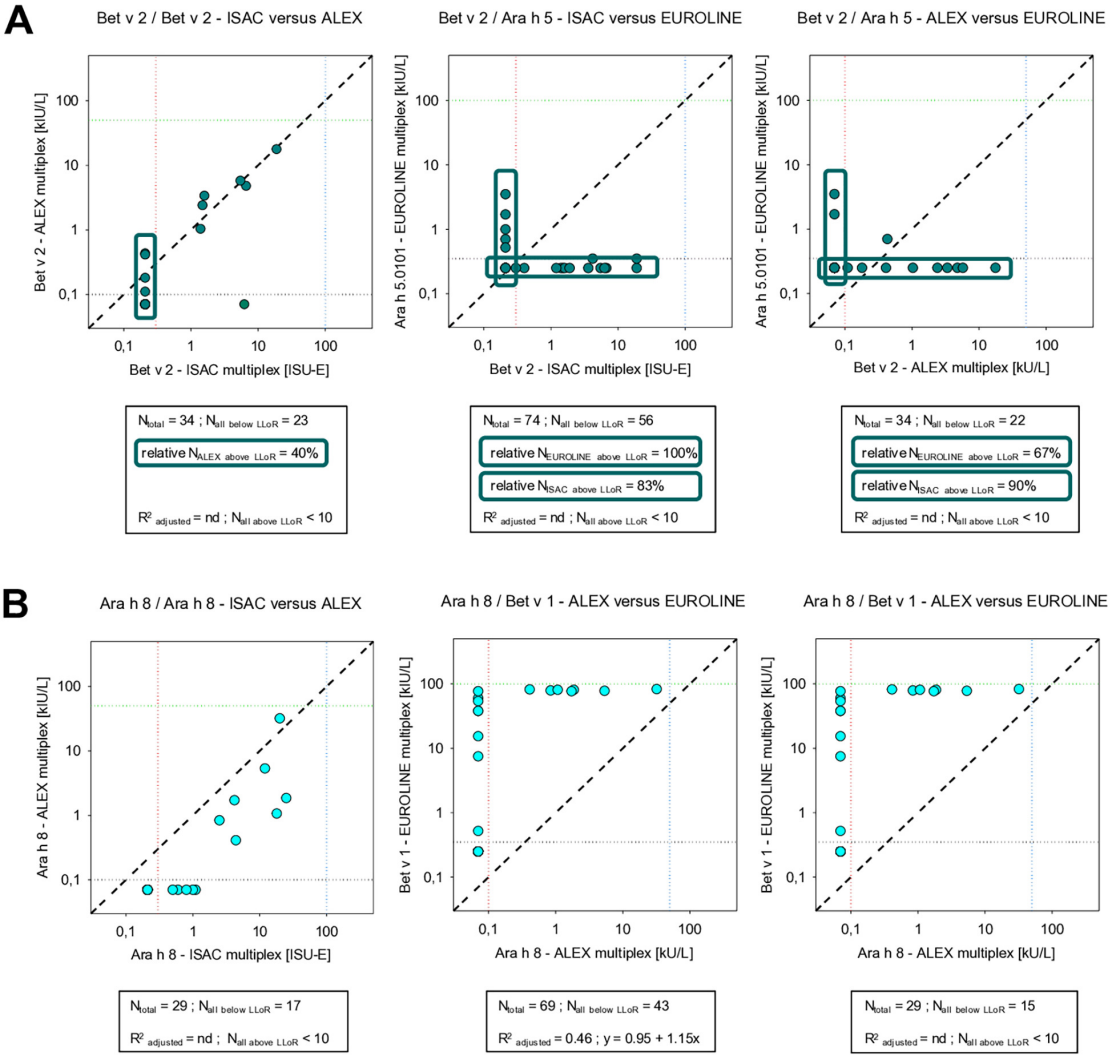
Correlation between concentrations of sIgE against some profilins and PR-10 proteins as detected by three multiplex assays: **(A)** Bet v 2 vs. Bet v 2 or Bet v 2 vs. Ara h 5 and **(B)** Ara h 8 vs. Ara h 8 or Ara h 8 vs. Bet v 1 (for an explanation of the meaning of the gridlines, see the respective information in Figure 6).

Surrogate allergen components did not correlate at all (peanut profilin Ara h 5 vs. birch profilin Bet v 2) or less adequately (peanut PR-10 protein Ara h 8 vs. birch PR-10 protein Bet v 1) with true peanut components (Figures 5A,B). As indicated, the same applies for peanut LTP Ara h 9.0201 vs. peach LTP Pru p 3.

#### 3.3.4 Cross-reactive carbohydrate determinants

**Figure 6:**
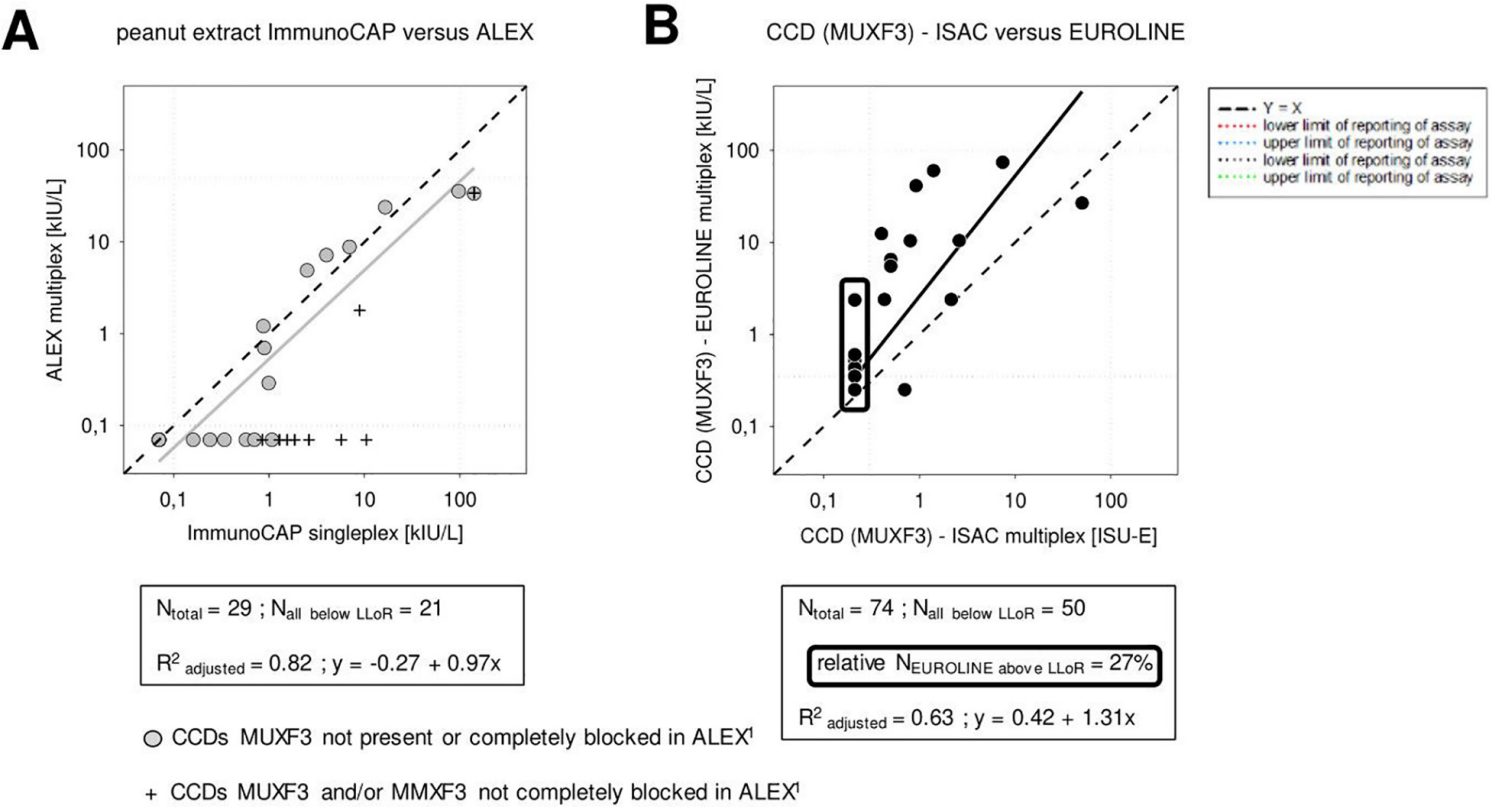
Correlation between concentrations of sIgE: **(A)** against peanut extracts as detected by the singleplex ImmunoCAP and multiplex ALEX^1^ assays and **(B)** against CCD (MUXF3) as detected by the ISAC and ALEX multiplex assays.

Application of a CCD blocker in ALEX demonstrated the frequent presence of peanut sIgE in combination with CCD sensitization for peanut whole extracts. Although CCD blocking in ALEX was effective, it was not always complete or consistent (Figure 6). The inconsistency was associated with the presence of IgE against Hom s lactoferrin. The sIgE results of CCD of ISAC vs. EUROLINE correlated adequately (Figure 6).

#### 3.3.5 Overall features

As a whole, the characteristics of peanut and CCD allergen components, as applied in the ISAC, ALEX, and EUROLINE panels, can also be classified based on subjective interpretations such as good, bad, and ugly features (Table 3).

**Table 3:** The good, bad, and ugly features of peanut and CCD allergen components as applied in the ISAC, ALEX, and EUROLINE panels.

Peanut component (PC)	Biochemical designation of PC	Classification of overall features	Description of overall features
*Arachis hypogaea*	WHO/IUIUS		
Ara h 1	Cupin (vicillin-type, 7S Globulin) storage protein	Good	Adequate analytical correlation between the ISAC, ALEX, and EUROLINE panels, although a mixture of Ara h 1 isoallergens in the ALEX panel seems to detect more patients compared to that in the ISAC and EUROLINE panels.
Ara h 2	Conlutin (2S albumin) storage protein	Good	Adequate analytical correlation between the ISAC, ALEX, and EUROLINE panels, although Ara h 2.0101 isoallergen in the ISAC panel seems to detect more patients than Ara h 2.0201 isoallergen in the EUROLINE panel.
Ara h 3	Cupin (legumin-type, glycinin, 11S globulin) storage protein	Good	Adequate analytical correlation between the ISAC, ALEX, and EUROLINE panels, although a mixture of Ara h 3 isoallergens in the ALEX panel seems to detect more patients compared to that in the ISAC and EUROLINE panels.
Ara h 5	Profillin	Ugly	Present and available only in the EUROLINE panel; no analytical correlation at all between the true profilin Ara h 5 of EUROLINE and the surrogate profilin Bet v 2 of ISAC and ALEX panels; limited consequences for clinical interpretation of results as profillins are associated with very low risk for severe allergic reactions.
Ara h 6	Conlutin (2S albumin) storage protein	Good	Adequate analytical correlation between the ISAC, ALEX, and EUROLINE panels, although recombinant Ara h 3 isoallergen in the EUROLINE panel seems to detect more patients compared to that in the ISAC and ALEX panels.
Ara h 7	Conlutin (2S albumin) storage protein	Good	Present and available only in the EUROLINE panel; adequate correlation between the 2S albumins Ara h 7 of EUROLINE and Ara h 2 of ISAC and ALEX panels; limited clinical consequences as no mono-sensitization toward Ara h 7 was observed.
Ara h 8	PR-10 protein, Bet v 1 family member	Ugly	Not available in the EUROLINE panel; limited correlation between the surrogate PR-10 protein Bet v 1 of EUROLINE panel and true PR-10 protein Ara h 8 of the ISAC and ALEX panels; limited consequences for clinical interpretation of results as PR-10 proteins are associated with a low risk for severe allergic reactions.
Ara h 9	Non-specific lipid transfer protein type 1	Bad	No adequate correlation between the EUROLINE panel containing Ara h 9.0201 isoallergen and ISAC and ALEX panels containing Ara h 9.010 isoallergen; significant clinical consequences if clinical studies are focused on the less relevant Ara h 9 isoallergen.
Ara h 15	Oleosin	Unknown	Present and available only in the ALEX^2^ panel; no comparison is possible with other surrogate oleosins.
CCD	Cross-reactive carbohydrate determinant	Good	Adequate analytical correlation between the CCD (MUXF3) of the ISAC and EUROLINE panels and, based on peanut extract results, adequate CCD (MUXF3) blocking in the ALEX panels.

## 4 Discussion

### 4.1 Impact of different analytical designs of multiplex assays

The assay characteristics of the multiplex assays investigated were diverse and the required sample volumes varied significantly. The consequence is that, for multiplex assays, the absolute amount of sIgE present in the aliquots varied and, when applied in the assays, had to deal with the binding capacity of coupled allergens to the solid phase in these assays. Singleplex IgE assays typically use high quantities of allergen (micrograms) to bind as much sIgE as possible in the presence of relatively high concentrations of specific immunoglobulin G (sIgG) against the same allergens. As multiplex assays use considerably lower quantities of allergen (for example, femtogram quantities for the ISAC panel), these assays have lower binding capacity for sIgE (14, 15). Because of this, multiplex assays are sensitive to competition with sIgG, especially if that sIgG blocks access to the allergen when the quantity of coupled allergen on the solid phase is limited. For ISAC, this has already been well established, while for EUROLINE and ALEX, these still need to be investigated (4).

As for singleplex assays, one challenge of multiplex assays relates to the quantitation of sIgE. International reference preparations (IRPs) are only available for total and not for sIgE (16). Heterologous interpolation from total IgE (kU/L) to allergen-sIgE (kU**_A_**/L) units in standard calibration schemes remains an important way to calculate the concentration of sIgE, both in singleplex (ImmunoCAP) and multiplex assays (EUROLINE and ALEX). In contrast, ISAC applies a homologous calibration. However, its manufacturer must still ensure that the respective units (ISU-E) are equivalent to an IRP (kU/L) and can quantitatively be compared to, for example, a singleplex assay (kU**_A_**/L). This is why ISAC concentrations are expressed in an ISAC standardized unit for sIgE (ISU-E). Moreover, in multiplex assays, the values of calibration points for sIgE against components, which are panallergens and can bind on different components within the assay, must be corrected for the phenomenon of cross-reactivity. This requires correction factors, of which the manufacturer must guarantee the quality and robustness.

Another challenge for the quantitation of sIgE and total IgE is the arrangement of the dynamic range of a multiplex assay. This is not defined by the reporting range but is related directly to the linearity and sensitivity of the applied detection technology. The dynamic range of a detector is defined as the difference between its background noise and saturation intensity. Near the background noise, the detector response should be as narrow and low as possible, ensuring that relatively low concentrations can be measured as precisely as possible. Above the saturation intensity, the detector response is constant, and thus different analytical response levels cannot be distinguished. Because of this, fluorescence spectroscopy, used as a detection technology in ISAC, has several advantages such as improved sensitivity and specificity, resulting in a wide concentration range in terms of logarithmic orders of concentration without sample dilution (Figure 1). Furthermore, fluorescence is capable by definition of achieving lower limits of detection and thus of quantitation and reporting, which offers the opportunity to use less sample material. This is important, especially when working with precious or limited-quantity sample materials such as in allergy diagnosis and management. This also explains why, in ISAC, less sample material is being applied compared to EUROLINE and ALEX, as both the latter detection technologies are based on colorimetry in combination with an enzyme. Colorimetry is limited to a considerably smaller range of logarithmic orders of measured concentrations compared to fluorescence. This clarifies why, in the EUROLINE analytical design, the assay shows a less steep or even a slightly flattened response curve (Figure 1) (11, 12). In general, the flattening of the response curve affects the analytical sensitivity in the higher concentration range. However, colorimetry does not hinder the impact of EUROLINE as the additional value of high concentrations of sIgE, in combination with multiplex assays to measure broad patterns of IgE-mediated sensitizations, is negligible anyway (11). The same applies to the ALEX versions and might also explain why the calibration range of sIgE in the ALEX versions ranges from 0.3 to only 50 kU**_A_**/L instead of 100 kU**_A_**/L, as for EUROLINE, or 100 ISU-E, as for ISAC.

While the EUROLINE and ISAC sIgE assays were qualified by their manufacturers as semi-quantitative assays, the ALEX panels were marketed as a quantitative assay for sIgE. There may be analytical reasons to qualify an assay as a quantitative or semi-quantitative one, but it may also be driven by regulatory issues, which are more strict and extensive for quantitative assays than for semi-quantitative ones. Because of commercial reasons, manufacturers may select the appropriate and cheapest regulatory obstacle if the impact on the clinical interpretations allows it.

The overall impact of the different analytical designs is that quantitative results of different multiplex assays are challenging to compare. The manufacturers involved may underline that such a quantitative comparison is meaningless if two of the multiplex assays are claimed to be semi-quantitative. However, in real-life practice, the results of multiplex assays are interpreted predominantly in the literature as being pure quantitative results (11, 12). Very rarely are the results presented in merely semi-quantitative classes. Therefore, a comparison of pure quantitative results has a value for real-life situations.

### 4.2 Impact of component origin and composition

In the multiplex assays reviewed, the exact origin of peanut allergen compounds regarding the isoallergen composition is not always equal, which might have some additional impact. For example, peanut sensitizations toward different allergen components lead to subpopulations that can be clinically distinguished based on geographical regions (17). The question is whether sensitizations toward the same peanut allergen component, but with a different isoallergen composition, also lead to further identifiable subpopulations.

#### 4.2.1 Storage protein components

The peanut components associated with severe allergic reactions are the cupin storage protein pair Ara h 1 and 3 and the 2S albumin storage protein pair Ara h 2 and 6 (18, 19). A distinct feature of Ara h 6 in the ISAC and ALEX^1^ panels and of Ara h 1 and Ara h 3 in the ALEX panels is the use of natural peanut allergen components. The presence of some subpopulations in this study was associated with the application of components originating from natural extracts (Figures 2A–C). These natural extracts likely consist of a mixture of isoallergens of the same storage protein components. The theoretical advantage of applying a mixture of isoallergens in an assay is that it covers a larger test menu of possible sources of IgE sensitization. This would support the idea that assay designs are less critical than the exact composition of allergen components. However, the subpopulation for Ara h 6 detected by the EUROLINE panel is associated with the use of a recombinant source as this panel applied a recombinant Ara h 6 isoallergen, whereas the ALEX^1^ and ISAC panels used a natural mixture (Figure 2D). ALEX^2^ also applied the recombinant Ara h 6 isoallergen, but in our study, the number of ALEX observations is dominated by those of ALEX^1^ (29 vs. 5 observations). The subpopulation for Ara h 2 detected by the ISAC panel is associated with the application of the specific Ara h 2.0101 isoallergen instead of Ara h 2.0201 by the other panels.

As indicated, the direct impact of measuring sIgE against the 2S albumin storage protein component Ara h 7 by the EUROLINE panel in this study is very restricted. No mono-sensitization toward Ara h 7 was observed. It was only found in combination with sensitization toward the 2S albumin storage protein components Ara h 2 and Ara h 6. This may also be the result of cross-reactions in the assays when measuring sIgE against 2S albumin storage proteins in general. This may call into question the additional value of measuring sIgE against Ara h 7. However, if Ara h 7 is not part of the EUROLINE panel, this information would and will not be accessible. The results of Ara h 2 vs. those of Ara h 7 also differed as the ALEX and ISAC panels both detected an Ara h 2 subpopulation, once more demonstrating the heterogeneous nature of specific IgE results for storage protein components.

Our results for sIgE against storage proteins are in agreement with the literature, where ISAC and ALEX were compared (12, 20–22). A critical note is that storage protein components obtained and purified from whole extracts may be contaminated with impurities. Therefore, the detection of subpopulations in our study could also be attributed to possible impurities of other components rather than to a mixture of isoallergens of the same storage protein components. It would be of interest to investigate the impact of using a mixture of recombinant components relative to purified components from natural extracts. Another critical note is that a peanut allergen component may contain glycans and thus CCD-like epitopes, such as for Ara h 1 (23). Especially for components of natural origin rather than recombinant origin, this is relevant (Table 2). This way, in the ALEX panel, by applying the CCD blocker, nAra h 1 subpopulations may be detected and misidentified as isoallergen specific. This merely underlines that adequate information regarding the exact nature of allergen components is important. The clinical impact of the detection of subpopulations by either using a natural mixture of isoallergens or by applying specific recombinant isoallergens is of great importance.

#### 4.2.2 Lipid transfer protein components

LTP components, in general, are associated with conflicting mild and severe allergic reactions, which may be influenced by the absence or presence of co-factors (19, 24). The fact that, in EUROLINE, another peanut LTP isoallergen is applied compared to ISAC and ALEX (Table 2) may explain the high responses in EUROLINE and the detection of Ara h 9 subpopulations. This would suggest that, in clinical practice, the focus should additionally be on sIgE against Ara h 9.0201 instead of only on Ara h 9.0101, and should at least be focused on a mixture of both sIgEs. In Mediterranean countries, the existence of specific Ara h 9.0101 and 9.0201 subpopulations was already indicated in 2009 (13). The underestimation of measuring Ara h 9 by ISAC also has been suggested previously (25), while in an ALEX study, the contribution of Ara h 9 in the *in vitro* diagnosis of peanut allergy was negligible (26), indirectly calling into question the clinical significance of sIgE against Ara h 9.0101 in these studies. This would confirm that the unclear clinical significance of Ara h 9 in some studies may also be due to the incomplete analytical focus regarding the isoallergen specificity of Ara h 9 in the assays applied. The meaning of Ara h 9.0201 also has been studied, specifying clinically relevant epitopes of Ara h 9.0201 in relation to other LTPs (27). Regrettably, in the same study, the investigators failed to point out that by using ISAC as a reference method for IgE against intact Ara h 9, they referred to a method based on intact Ara h 9.0101 and compared it with a method based on Ara h 9.0201 epitopes. Once more, transparency and adequate information are the key factors in the interpretation of multiplex allergy results.

The observation that, in our Dutch study, Ara h 9.0201 in the EUROLINE panel did not correlate adequately with peach LTP in the other assays may confirm that between different LTPs, cross-reactivity has not been conserved uniformly during evolution and is expressed differently per region depending on the route of exposure (28). The recent development of an extensive LTP EUROLINE panel containing 28 different recombinant LTPs is of special interest assuming that it also encompasses the Ara h 9.0201 isoallergen (29). This study showed in a northern Spanish patient cohort with LTP sensitizations, 70% of the patients studied were sensitized against the assumed peanut LTP Ara h 9.0201 isoallergen. Unfortunately, although the origins of the LTPs are specified, the isoallergen and variant of the enclosed LTPs in the LTP EUROLINE panel were only partially specified. Finally, and to underline the complexity of LTP relevancy in the immunological playing field, the clinical relevancy is not only determined by polyclonal IgE peptide recognition but also by IgG4 recognition of Ara h 9 (30). Therefore, the absence or presence of co-factors may not be the sole element to explain the conflicting reactions associated with LTPs.

#### 4.2.3 Surrogate components

The value of measuring sIgE against the profilin protein component Ara h 5 and that of the PR-10 Bet v 1 in the same EUROLINE panel seems to have very limited meaning. After all, in this study, sIgE against Ara h 5 did not correlate at all with that of profilin Bet v 2 in other panels, whereas measuring sIgE against Bet v 1 overestimated the PR-10 sensitization toward Ara h 8. Moreover, in Europe, sensitizations toward Ara h 5 and Ara h 8 are mainly grass and birch pollen-related, respectively. As both proteins are considered to be labile to heat and digestion and since peanuts are generally not consumed raw in Europe, it may be expected that the relevant clinical symptoms are limited (18, 19, 31).

#### 4.2.4 Cross-reactive carbohydrate determinants

One of the unique features of the ALEX assay concept is the use of the CCD inhibitor in the analytical procedure. CCDs are carbohydrate residues of glycoproteins that can result in the generation of CCD-sIgE antibodies without any clinical significance. Their presence and thus detection in blood hinders the clinical interpretation of analytical sIgE results if the targeted allergen contains extracts of interest or if allergen components are CCD-containing glycoproteins (9, 32). In at least 30% of the patients subjected to *in vitro* allergy diagnosis, CCD-sIgE antibodies can be detected (9, 33). As indicated, the CCD inhibitor contains CCDs based on the carbohydrate core MUXF3.

While ALEX^1^ still incorporated component Ana c 2 as the MUXF3-containing glycoprotein to monitor the efficiency of the CCD inhibitor, ALEX^2^ does not incorporate Ana c 2. The consequence is that ALEX^1^ directly monitors the inhibition by the CCD inhibitor, MUXF3-HSA, while ALEX^2^ cannot, leaving one in the dark regarding the efficiency of CCD inhibition. In contrast to this, ALEX^1^ and ALEX^2^ both also include recombinant glycoprotein Hom s lactoferrin expressed in rice, which may contain CCDs that are 30% based on MUXF3 and 50% on another CCD-like residue, namely MMXF3 (34). MMXF3 indicates the presence of two terminal mannoses instead of one terminal mannose to the proximal mannose as in MUXF3. Therefore, the Hom s lactoferrin results cannot completely replace Ana c 2 as a monitor of CCD inhibition by MUXF3-HSA. In addition, lactoferrin from cow milk, and the potential trigger of milk-based IgE and IgG CCD sensitizations in humans, contains CCDs based on slightly different carbohydrate cores than MUXF3 and MMXF3 (35). Furthermore, certain pollen-based CCD sensitizations may vary in specificities toward even more carbohydrate cores (36). This merely demonstrates the potentially heterogeneous nature of CCD sensitizations, which is not limited to sensitization against MUXF3 and therefore not always reliably measured.

The CCD complexity is illustrated in the study by Aumayr et al. with a small number of samples (*N* = 15), which all were positive for sIgE using ImmunoCAP. MUXF3 ALEX showed 100% positive results for sIgE against Hom s lactoferrin and 40% positive results against Ana c 2 prior to CCD blocking by MUXF3-HSA (37). This might justify the exclusion of Ana c 2 in the ALEX^2^ version compared to the ALEX^1^ version but also might underline that the CCD inhibitor may not fully block all sIgE CCD sensitizations detected by Hom s lactoferrin. After all, Hom s lactoferrin also contains MMXF3. Our results regarding CCDs also illustrate that the blocking was not complete or inconsistent, especially when IgE against Hom s lactoferrin was present. Indeed, in the same study by Aumayr et al., the blocking rate did not always reach 100%, although it cannot be excluded that the absolute capacity of the CCD inhibitor was insufficient under the conditions applied. To optimize the blocking process, the instructions for the use of ALEX^2^ describe two different analytical protocols, one applying the CCD inhibitor during the incubation of the sample on the array and the other applying the inhibitor before the incubation on the array.

The use of a CCD inhibitor in combination with ALEX might have been born of necessity, as cellulose is known to contain small quantities of CCD itself (9, 16, 38). Therefore, applying nitrocellulose as the basis for the solid phase of ALEX automatically implies the use of a CCD inhibitor because of analytical reasons. Predictable and important, it has the benefit of eliminating a broad factor that complicates the clinical interpretation of CCD-affected results.

#### 4.2.5 Subpopulations

For all panallergens, we detected in our correlation studies several subpopulations, which were not excluded in the calculation of correlations and thus affected the correlation coefficients. Although one can argue about how to calculate a correlation and also about the exact definition of a subpopulation, it is clear that subpopulations do exist. The true or even false identification of subpopulations can mislead clinicians with clear consequences for the patient. Therefore, based on these evolving insights, the exact nature of detected subpopulations or the reason for detecting those apparent subpopulations should be the subject of future research when studying multiplex assays.

## 5 Conclusion and final remarks

Multiplex IgE peanut panels correlated adequately for the clinically relevant Ara h storage proteins and less adequately for the clinically relevant LTP Ara h 9. Furthermore, the panels indicated that the nature of CCD sensitization was heterogeneous. Based on this, we classified the characteristics of the peanut allergen components on their good, bad, and even ugly features. Besides differences in analytical design, the panels did not have the same component origin and isoallergen composition to bind and detect sIgE, which significantly affected the correlations and detection of subpopulations of patients. In particular, for the LTP Ara h 9, this was very striking and needs further in-depth evaluation. Therefore, knowledge of the origin and respective isoallergen specification of the peanut allergen components, including the exact CCD composition, is essential. Together with that of the variants, this knowledge should be documented more adequately in scientific studies and in the respective instructions for the use of multiplex allergy assays. This will refine and thus improve the rationalization of the clinical decision-making process in peanut allergy diagnosis and treatment, despite the fact that significant steps have already been taken in recent years.

Bernardini et al. indicated that multiplex panels are harmful if clinicians lack adequate knowledge of molecular allergy (39). However, if manufacturers do not properly inform clinicians about the composition of their assays, clinicians will not be able to act properly. Nevertheless, we should also be aware that, although transparency accelerates the extent of our knowledge, a greater understanding will not simplify the interpretation of the results of multiplex assays in allergy, but will merely illustrate its complexity.

Based on this study, we would like to encourage manufacturers of multiplex allergy assays to present a full specification of the allergen components involved. Currently, the origins are always specified, but the isoallergen and variant are seldom specified by manufacturers, despite their attempt to comply with regulations and directives of the North American Food and Drug Administration (FDA) and the Chinese and European counterpart organizations. In Europe, the current transition from the In-Vitro Medical Device Directive (IVDD) to the In-Vitro Diagnostic Regulation (IVDR), which introduces more stringent regulation and focuses on, among others, more adequate descriptions of assay characteristics, means this kind of documentation may be very relevant. Therefore, such a transition deserves some additional attention from manufacturers of not only multiplex allergy assays but also singleplex assays.

A limitation of our study is that the presence or absence of subpopulations might also be an outcome of statistics, as we used a relatively large population of patients (*N* = 74) to compare directly two panels but used a relatively small population of patients (*N* = 34) to compare three panels (Supplementary Material S1). Moreover, as several peanut allergen components play different regional roles in peanut allergy worldwide (13, 17–19, 24), our study reflects the situation in the south of the Netherlands. While there may be some other limitations of our study, the message remains that a lack of information may affect the decisions of clinicians.

## Data Availability

The raw data supporting the conclusions of this article will be made available by the authors, without undue reservation.

## References

[1] World Allergy Organization (WAO), Canonica GW Ansotegui IJ Pawankar R Schmid-Grendelmeier P van Hage M A WAO - ARIA - GA2LEN consensus document on molecular-based allergy diagnostics. World Allergy Organ J. (2013) 6:17. 10.1186/1939-4551-6-17 24090398 PMC3874689

[2] Jakob T Forstenlechner P Matricardi P Kleine-Tebbe J. Molecular allergy diagnostics using multiplex assays: methodological and practical considerations for use in research and clinical routine: part 21 of the series molecular allergology. Allergo J Int. (2015) 24:320–32. 10.1007/s40629-015-0087-8 27069843 PMC4792369

[3] World Allergy Organization (WAO), Steering committee authors; review panel members. A WAO - ARIA - GA2LEN consensus document on molecular-based allergy diagnosis (PAMD@): update 2020. World Allergy Organ J. (2020) 13:100091. 10.1016/j.waojou.2019.100091 32180890 PMC7062937

[4] Keshavarz B Platts-Mills TAE Wilson JM. The use of microarray and other multiplex technologies in the diagnosis of allergy. Ann Allergy Asthma Immunol. (2021) 127:10–8. 10.1016/j.anai.2021.01.003 33450398 PMC9107268

[5] Quan PL Sabaté-Brescó M D'Amelio CM Pascal M García BE Gastaminza G Validation of a commercial allergen microarray platform for specific immunoglobulin E detection of respiratory and plant food allergens. Ann Allergy Asthma Immunol. (2022) 128:283–90.e4. 10.1016/j.anai.2021.11.019 34863952

[6] World Health Organization (WHO), WHO IUIS Allergen Nomenclature Sub-Committee, Pomés A Davies JM Gadermaier G Hilger C WHO/IUIS allergen Nomenclature: providing a common language. Review Mol Immunol. (2018) 100:3–13. 10.1016/j.molimm.2018.03.003 29625844 PMC6019191

[7] Coreon, Committee on Regulation of Health Research. (2024). Available online at: https://www.coreon.org/about-coreon/ (accessed September 17, 2024).

[8] Hornung RW Reed LD. Estimation of average concentration in the presence of non-detectable values. Appl Occup Envir Hyg. (1990) 5:46–51. 10.1080/1047322X.1990.10389587

[9] Altmann F. Coping with cross-reactive carbohydrate determinants in allergy diagnosis. Allergo J Int. (2016) 25:98–105. 10.1007/s40629-016-0115-3 27656353 PMC5016538

[10] Platts-Mills TA Hilger C Jappe U van Hage M Gadermaier G Spillner E Carbohydrate epitopes currently recognized as targets for IgE antibodies. Allergy. (2021) 76:2383–94. 10.1111/all.14802 33655520 PMC8489568

[11] Hoang JA Celik A Lupinek C Valenta R Duan L Dai R Modeling the conversion between specific IgE test platforms for nut allergens in children and adolescents. Allergy. (2021) 76:831–41. 10.1111/all.14529 32738829

[12] Platteel ACM van der Pol P Murk JL Verbrugge-Bakker I Hack-Steemers M Roovers THWM A comprehensive comparison between ISAC and ALEX2 multiplex test systems. Clin Chem Lab Med. (2022) 60:1046–52. 10.1515/cclm-2022-0191 35470638

[13] Krause S Reese G Randow S Zennaro D Quaratino D Palazzo P Lipid transfer protein (Ara h 9) as a new peanut allergen relevant for a Mediterranean allergic population. J Allergy Clin Immunol. (2009) 124:771–8.e5. 10.1016/j.jaci.2009.06.008 19665774

[14] Lupinek C Wollmann E Baar A Banerjee S Breiteneder H Broecker BM. Advances in allergen-microarray technology for diagnosis and monitoring of allergy: the MeDALL allergen-chip. Methods. (2014) 66:106–19. 10.1016/j.ymeth.2013.10.008 24161540 PMC4687054

[15] Van Hage M Hamsten C Valenta R. ImmunoCAP assays: pros and cons in allergology. J Allergy Clin Immunol. (2017) 140:974–77. 10.1016/j.jaci.2017.05.008 28552762

[16] Kleine-Tebbe J Poulsen LK Hamilton RG. Quality management in IgE-based allergy diagnostics. J Lab Med. (2016) 40:81–96. 10.1515/labmed-2016-0013

[17] Vereda A van Hage M Ahlstedt S Ibañez MD Cuesta-Herranz J van Odijk J Peanut allergy: clinical and immunologic differences among patients from 3 different geographic regions. J Allergy Clin Immunol. (2011) 127:603–7. 10.1016/j.jaci.2010.09.010 21093026

[18] Palladino C Breiteneder H. Peanut allergens. Mol Immunol. (2018) 100:58–70. 10.1016/j.molimm.2018.04.005 29680589 PMC7060077

[19] Randazzese SF Panasiti I Caminiti L Catamerò F Landi M De Filippo M Current state and advances in desensitization for peanut allergy in pediatric age. Pediatr Allergy Immunol. (2024) 35(4):e14127. 10.1111/pai.14127 38646959

[20] Heffler E Puggioni F Peveri S Montagni M Canonica GW Melioli G. Extended IgE profile based on an allergen macroarray: a novel tool for precision medicine in allergy diagnosis. World Allergy Organ J. (2018) 11:7. 10.1186/s40413-018-0186-3 29743964 PMC5918992

[21] Scala E Caprini E Abeni D Meneguzzi G Buzzulini F Cecchi L A qualitative and quantitative comparison of IgE antibody profiles with two multiplex platforms for component-resolved diagnostics in allergic patients. Clin Exp Allergy. (2021) 51:1603–12. 10.1111/cea.14016 34523179

[22] Sonneveld LJH Emons JAM Arends NJT Landzaat LJ Veenbergen S Schreurs MWJ. ALEX versus ISAC multiplex array in analyzing food allergy in atopic children. Clin Mol Allergy. (2022) 20:10–6. 10.1186/s12948-022-00177-w 36030246 PMC9419344

[23] Kolarich D Altmann F. N-Glycan analysis by matrix-assisted laser desorption/ionization mass spectrometry of electrophoretically separated nonmammalian proteins: application to peanut allergen Ara h 1 and olive pollen allergen ole e 1. Anal Biochem. (2000) 285:64–75. 10.1006/abio.2000.4737 10998264

[24] Miralles-Lopez JC Carbonell-Martínez A Zamarro-Parra S Navarro-Garrido C Escudero-Pastor AI Boulaich M Clinical and serological characteristics of patients allergic to LTP. Allergol Immunopathol (Madr). (2024) 52(4):9–14. 10.15586/aei.v52i4.1074 38970259

[25] Goikoetxea MJ D'Amelio CM Martínez-Aranguren R Gamboa P Garcia BE Gómez F Is microarray analysis really useful and sufficient to diagnose nut allergy in the Mediterranean area? J Investig Allergol Clin Immunol. (2016) 26:31–9. 10.18176/jiaci.0005 27012014

[26] Aytekin ES Soyer O Sahiner UM Wieser S Lupinek C Sekerel BE. Diagnostic accuracy of the ALEX2 test in peanut-sensitized children. Clin Exp Allergy. (2023) 53:1041–44. 10.1111/cea.14350 37246604

[27] Kronfel CM Cheng H McBride JK Nesbit JB Krouse R Burns P IgE epitopes of Ara h 9, Jug r 3, and Pru p 3 in peanut-allergic individuals from Spain and the US. Front Allergy. (2023) 3:1090114. 10.3389/falgy.2022.1090114 36698378 PMC9869384

[28] Asero R Pravettoni V Scala E Villalta D. Lipid transfer protein allergy: a review of current controversies. Clin Exp Allergy. (2022) 52:222–30. 10.1111/cea.14049 34773669

[29] Balsells-Vives S Flügge U Brix B Weimann Y Peralta T San Bartolomé C Improving *in vitro* detection of sensitization to lipid transfer proteins: a new molecular Multiplex IgE assay. Mol Nutr Food Res. (2023) 67:e2200906. 10.1002/mnfr.202200906 37195823

[30] Sánchez-Ruano L Fernández-Lozano C Ferrer M Gómez F de la Hoz B Martínez-Botas J Differences in linear epitopes of Ara h 9 recognition in peanut allergic and tolerant, peach allergic patients. Front Allergy. (2022) 3:896617. 10.3389/falgy.2022.896617 35935018 PMC9352880

[31] Lange L Beyer K Kleine-Tebbe J. Benefits and limitations of molecular diagnostics in peanut allergy: part 14 of the series molecular allergology. Allergo J Int. (2014) 23:158–63. 10.1007/s40629-014-0019-z 26120527 PMC4479434

[32] Sinson E Ocampo C Liao C Nguyen S Dinh L Rodems K Cross-reactive carbohydrate determinant interference in cellulose-based IgE allergy tests utilizing recombinant allergen components. PLoS One. (2020) 15:e0231344. 10.1371/journal.pone.0231344 32324770 PMC7179882

[33] Mari A. Ige to cross-reactive carbohydrate determinants: analysis of the distribution and appraisal of the *in vivo* and *in vitro* reactivity. Int Arch Allergy Immunol. (2002) 129:286–95. 10.1159/000067591 12483033

[34] Fujiyama K Sakai Y Misaki R Yanagihara I Honda T Anzai H N-linked glycan structures of human lactoferrin produced by transgenic rice. Biosci Biotechnol Biochem. (2004) 68:2565–70. 10.1271/bbb.68.2565 15618628

[35] Karav S German JB Rouquié C Le Parc A Barile D. Studying lactoferrin N-glycosylation. Int J Mol Sci. (2017) 18:870–84. 10.3390/ijms18040870 28425960 PMC5412451

[36] Yokoi H Yoshitake H Matsumoto Y Kawada M Takato Y Shinagawa K Involvement of cross-reactive carbohydrate determinants-specific IgE in pollen allergy testing. Asia Pac Allergy. (2017) 7:29–36. 10.5415/apallergy.2017.7.1.29 28154803 PMC5287067

[37] Aumayr M Forstenlechner P Kofler H. Blocking CCD specific IgE antibodies in a multiplex environment. Clin Transl Allergy. (2018) 8(Suppl 1):P54. 10.1186/s13601-018-0193-z

[38] Hemmer W Altmann F Holzweber F Gruber C Wantke F Wöhrl S. ImmunoCAP cellulose displays cross-reactive carbohydrate determinant (CCD) epitopes and can cause false-positive test results in patients with high anti-CCD IgE antibody levels. J Allergy Clin Immunol. (2018) 141:372–81.e3. 10.1016/j.jaci.2017.04.028 28506851

[39] Italian Society of Pediatric Allergy and Immunology, Allergy Diagnostic Commission, Bernardini R Arasi S Barni S Caimmi D Multiparametric or multiplex systems in allergy diagnostics. Ital J Pediatr Allergy Immunol. (2024) 38:17–21. .org/10.53151/2531-3916/2024-463

