# Supplementary figures and images for: Multiplex IgE peanut panels: a critical appraisal of assay designs and the good, the bad, and the ugly features of the applied allergen components
Source: Front Allergy. 2025 Jun 2;6:1515294. doi: 10.3389/falgy.2025.1515294 (PMC12171301; doi:10.3389/falgy.2025.1515294)

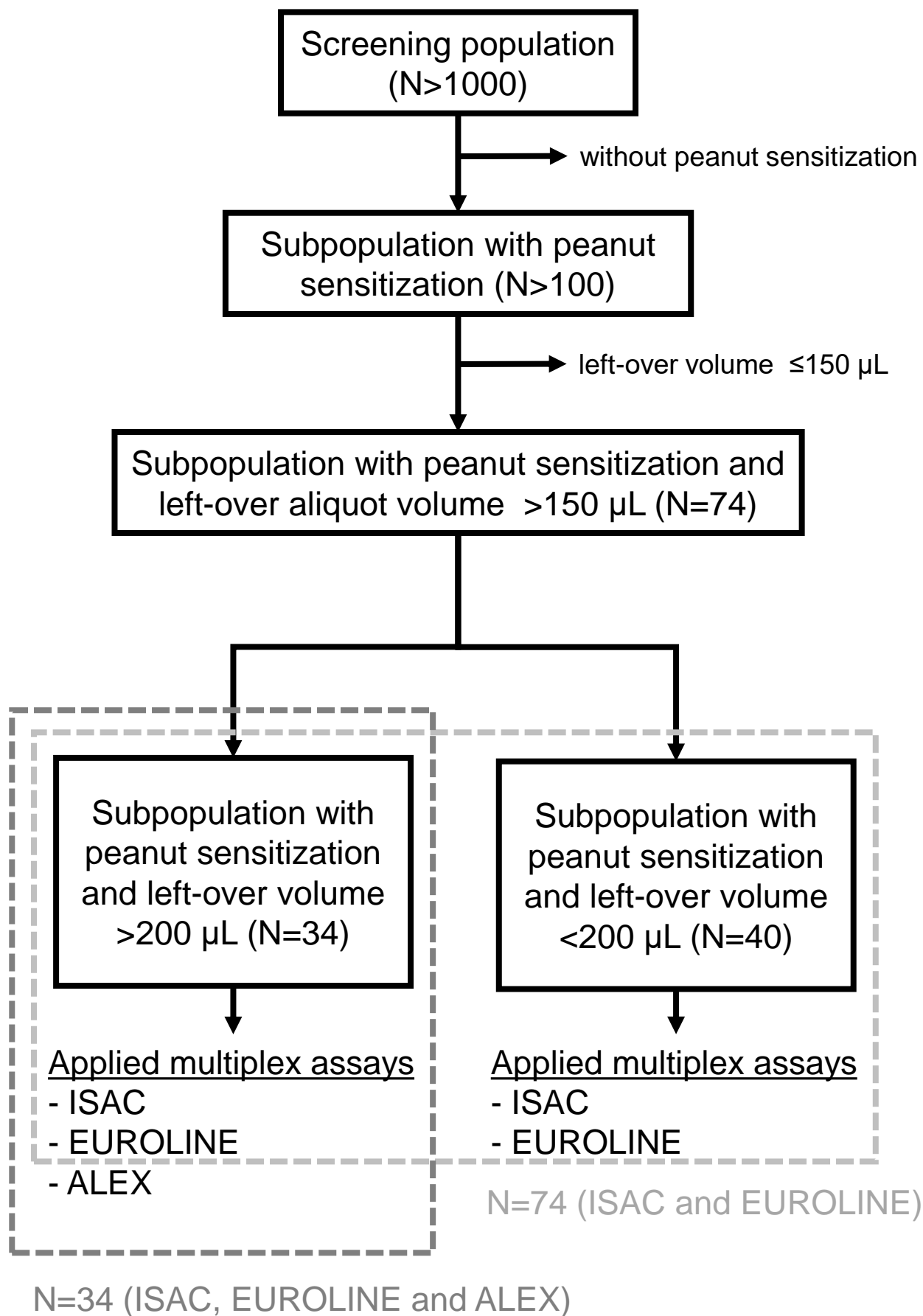

Supplement: Supplementary file 1 [file Datasheet1.pdf]
